# Uncontrolled Hypertension, Treatment, and Predictors among Hypertensive Out-Patients Attending Primary Health Facilities in Johannesburg, South Africa

**DOI:** 10.3390/healthcare11202783

**Published:** 2023-10-20

**Authors:** Amaziah Makukule, Perpetua Modjadji, Ntevhe Thovhogi, Kabelo Mokgalaboni, Andre Pascal Kengne

**Affiliations:** 1 Department of Public Health, School of Health Care Sciences, Sefako Makgatho Health Sciences University, 1 Molotlegi Street, Ga-Rankuwa 0208, South Africa; 2 Non-Communicable Disease Research Unit, South African Medical Research Council, Cape Town 7505, South Africa; 3 Department of Life and Consumer Sciences, College of Agriculture and Environmental Sciences, University of South Africa, Florida Campus, Roodepoort 1709, South Africa

**Keywords:** uncontrolled hypertension, treatment, predictors, primary health care facilities, out-patients, South Africa

## Abstract

Hypertension is a poorly controlled risk factor for cardiovascular disease in South Africa, particularly among patients receiving care in the public sector who are mostly from low socioeconomic backgrounds. This cross-sectional study investigated uncontrolled hypertension, treatment, and predictors among hypertensive out-patients attending primary health care facilities in Johannesburg, South Africa. The WHO STEPwise approach to the surveillance of non-communicable diseases was used to collect data, including sociodemographic and lifestyle factors, health status, and measurements for anthropometry and blood pressure along with self-reported adherence to treatment, estimated through the general medication adherence scale. Uncontrolled hypertension was defined as systolic blood pressure ≥140 mmHg and/or diastolic blood pressure ≥90 mmHg in diagnosed patients receiving anti-hypertensive treatment. Overweight and obesity were defined as a body mass index ≥25 and ≥30 kg/m^2^, respectively. Logistic regression models were used to assess the predictors of uncontrolled hypertension. Four hundred (*n* = 400) hypertensive out-patients (mean age: 50 ± 8 years) participated in this study, with most living in poor sociodemographic environments. The prevalence rate of uncontrolled hypertension was 57%. Obesity (62% vs. 42%, *p* ≤ 0.0001), salt consumption (90% vs. 55%, *p* ≤ 0.0001), alcohol intake (42% vs. 19%, *p* ≤ 0.0001), a smoking habit (23% vs. 4%, *p* ≤ 0.0001), alternative medicine use (51% vs. 40%, *p* = 0.043), and comorbidities (64% vs. 36%, *p* ≤ 0.0001) were higher in the uncontrolled group than the controlled group, whereas the prevalence of physical activity (38% vs. 15%, *p* ≤ 0.0001) was high in the controlled group vs. the uncontrolled. Overall, 85% of the patients moderately adhered to treatment, only 2% exhibited high adherence, and 13% demonstrated low adherence; over half of the patients received tri-therapy treatment. The predictors of uncontrolled hypertension are a number of prescribed antihypertensive therapies [adjusted odds ration = 2.39; 95% confidence interval: 1.48–3.87], treatment adherence [0.46; 0.21–0.97], salt consumption [28.35; 7.87–102.04], physical activity [0.22; 0.13–0.37], current alcohol use [2.10; 1.22–3.61], and current cigarette smoking [4.79; 1.88–12.18]. The high prevalence of uncontrolled hypertension in this study suggests a need to optimize prescriptions, adherence to BP-lowering medications, and lifestyle modifications. The management of comorbidities such as diabetes could offer considerable benefits in controlling blood pressure.

## 1. Introduction

Hypertension is a leading contributor to the global burden of non-communicable disease (NCD), and it disproportionately affects low- and middle-income countries (LMICs) [1,2], where awareness, detection, treatment, and control remain largely suboptimal [3,4]. A systematic review on the burden of undiagnosed hypertension in Sub-Saharan Africa reported hypertension prevalence ranging between 15% and 70%, with a pooled prevalence of 30% (27–34%). Of those with hypertension, 7% to 56% (pooled prevalence: 27%; 95% [95%CI: 23–31%]) were aware of their hypertensive status before the surveys. Overall, 18% (95%CI: 14–22%) of individuals with hypertension were receiving treatment, and only 7% (95%CI: 5–8%) had controlled blood pressure [5]. This might be due to non-adherence to antihypertensive treatment, implicated as one of the major contributing factors to uncontrolled hypertension [6,7,8,9], in addition to inadequate treatment, patients’ self-management practices [10,11], as well as sociodemographic status and disease profile [8,12]. Uncontrolled hypertension (i.e., defined as blood systolic blood pressure (BP) ≥ 140 mmHg and/or diastolic BP ≥ 90 mmHg in patients taking anti-hypertensive treatment) [13] predisposes an individual to myocardial infarction, heart failure, stroke, and kidney disease, ultimately increasing the risk of all-cause and cardiovascular disease mortality [14,15]. It is of enormous public health importance to adequately control hypertension to prevent cardiovascular events and increase the life expectancy of individuals living with chronic hypertension [16].

Hypertension is a common NCD across South African population groups, accompanied by comorbidities [17,18,19] and responsible for premature adult mortality [20,21]. To date, the prevalence of hypertension in the country is estimated between 21% and 77.9% [22,23], with 19.0% to 56.0% controlled [24,25]. Included in the South African guidelines on prevention and treatment of hypertension are recommended treatments and compliance for optimal management [26], yet uncontrolled hypertension (13.5% to 75.5%) is on the rise [3,4,13]. This could be partly due to the fact that a large proportion (85%) of South Africans have no private health insurance [27] and continue to use public PHC facilities that are accessible but compounded by a shortage of equipment and staff and medication, with challenges in the procurement process [28,29,30]. Access to first-line treatment is enabled by the Centralised Chronic Medicine Dispensing and Distribution Programme (CCMDD) in South Africa [31]. Currently, endeavours are underway to create the National Health Insurance (NHI) programme aimed at addressing inequalities that hamper access to comprehensive healthcare [32], in line with the World Health Organization on universal health insurance in LMICs [33] and sustainable development goals (SDG 3) for the United Nations on good health and wellbeing [34]. As there are few and local studies on uncontrolled hypertension in our region [3,4,13], this current study investigated uncontrolled hypertension, treatment, and predictors among hypertensive out-patients attending primary health care (PHC) facilities in Johannesburg, South Africa. 

## 2. Methods and Materials

### 2.1. Study Design and Framework

A cross-sectional study was conducted among hypertensive out-patients from February 2021 to February 2022. The concept of the study, data collection, and analysis were anchored on a conceptual framework for managing modifiable risk factors for cardiovascular diseases [35], suggesting a cluster of modifiable cardio-metabolic risk factors that precede cardiovascular diseases, including hypertension. Ultimately, these modifiable lifestyle risk factors predispose an individual to cardio-metabolic conditions, among urbanization, sociodemographic, and socio-cultural factors [35]. We used a 22-item checklist for cross-sectional studies reported through STROBE, Strengthening the Reporting of Observational Studies in Epidemiology [36].

### 2.2. Study Setting and Population

This study is set in PHC facilities located in the City of Johannesburg Metropolitan Municipality (COJ). COJ forms part of Gauteng, one of the nine provinces in South Africa, and has an approximated population of 16.1 million people [37]. There are 125 PHC facilities at COJ providing various services from maternal and child health care, chronic health, management of minor ailments, to emergency care. Various research from the Sefako Makgatho Health Sciences University (SMU) in South Africa has been conducted in COJ. 

### 2.3. Sample Size and Sampling

From the few studies conducted locally, the prevalence of uncontrolled hypertension is estimated between 13.5% and 75.5%, which we considered to estimate a minimum representative sample [3,13]. Applying the formula N = (Z1 − α)2 × P (1 − P)/D2 [4], a minimum sample of 334 was calculated, which was further confirmed through Rao software [38], estimating a minimum sample of 365 through an average population of 7250 chronic hypertensive patients visiting the facilities per month [39]. A buffered (10%) sample of 400 chronic hypertensive out-patients was obtained after excluding two questionnaires that had over 10% missing data. We resorted to convenience sampling to purposively select participants from the five PHC facilities in COJ based on the largest headcount after being unable to randomize sampling due to disorganized long queues in the facilities.

Following ethics approval and permission from the relevant bodies to conduct this study, we (i.e., main researcher and two trained research assistants) started with the recruitment of participants at various facilities with the help of the facility managers. During recruitments, the main researcher informed the patients on the purpose and procedures of the study, as well as the plans for data collection, in addition to distributing information leaflets written in a local language (isiZulu). Patients who met the inclusion criteria and who were receiving care at the selected facilities participated in the study. Eligible participants were chronic hypertensive out-patients enrolled in the CCMDD programme; aged ≥ 30 years; on two or more anti-hypertension drugs for more than six months, defined as a diuretic, calcium-channel blocker, an ACE inhibitor or angiotensin receptor blocker, alpha-blocker, vasodilator, or β-blocker able; residing in COJ; and willing to provide written consent. Any chronic patient with conditions contrary to these criteria was excluded from the study. 

### 2.4. Data Collection and Tools

#### 2.4.1. Sociodemographic Status and Related Factors

The WHO STEPwise questionnaire was modified, validated, and administered to the participants by the research team members [40]; additionally, the collecting of additional information on socio-demographic and contributing factors to uncontrolled hypertension was undertaken [41,42,43]. Data were collected on socio-demographic factors, anthropometric and physiological measurements, health behaviours such as a history of smoking cigarettes and alcohol consumption, physical activity, as well as a family history of NCD. Forward and backward translation of the questionnaire between isiZulu (one of the 11 local languages in South Africa) and English was done by an independent translator conversant with the two languages and confirmed by the main researcher who is isiZulu speaking. Thereafter, a pilot study was conducted among the few selected out-patients who did not form a part of the main study to pre-test the questionnaire and the feasibility of this study in the smallest facility. Content, construct, and face validity were ensured by two experts in the NCD field. The results from the pilot study showed that the questionnaire had relevant content and constructs and was easily arranged. The results of the pilot study were excluded from the analysis of the main study. 

#### 2.4.2. Anthropometric and Physiological Measurements

Participants’ weights were measured using a well-calibrated electronic scale (Elektra DLK Sports Electronic Body Stat Scale), while heights were measured using a stadiometer, both recorded to one decimal point and used to determine body mass index (BMI). Waist (WC) and hip (HC) circumferences were measured in centimetres using a non-stretchable measuring tape. From the anthropometric measurements, BMI values were used to categorize underweight (<18.9 kg/m^2^), normal weight (=19–24.9 kg/m^2^), overweight (≥25–29.9 kg/m^2^) and obesity (≥30 kg/m^2^) [44], while waist–hip ratio (WHR) and waist-to-height ratio (WHtR) were computed. All the measurements were guided by the World Health Organization’s (WHO) recommendations [44,45]. Blood pressure measurements were taken on the same day during consultation, measured using a sphygmomanometer (manufactured by Braun), and recorded as systolic (SBP) over diastolic (DBP) in millimetres of mercury (mmHg). From the medical files of each patient, additional blood pressure measurements from the last three different visits were also recorded in the questionnaire, as well as co-existing conditions (i.e., comorbidities).

#### 2.4.3. Operational Definitions

*Uncontrolled hypertension* was defined as SBP/DBP ≥ 140/90 mmHg in individuals who had been taking prescribed antihypertensive medications for at least six months, making any level below this controlled hypertension [46,47]. *Adherence to treatment* was determined through the adopted general medication adherence scale (GMAS) [48,49,50], obtained via asking 10 questions about compliance with treatment and scheduled appointments. Responses were “yes”, scored zero; “sometimes”, scored one point; or “no”, scored two points, with a total range from a minimum score of zero to a maximum score of 20 points. Scores between zero to nine, which is below 50%, were defined as low adherence; scores between 10 and 15, which is 50% to 79%, were defined as moderate adherence; high adherence was defined at scores between 16 and 20, which is (≥80%). Chronic disease coexisting with hypertension is a *comorbidity*, and the presence of comorbidity was indicated with “no” or “yes” [51]. A participant was considered *physically active*, categorized as “yes”, when meeting requirements for physical activity at least 30 min a day for 5 days a week, according to the Centers for Disease Control and Prevention (CDC), and “no” indicated being physically inactive [52]. *Current alcohol use* was defined as more than one unit and more than two units of alcohol/day for females and males, respectively, and alcohol use was categorized as “yes”, while not using alcohol was categorized as “no” [53]. *Current cigarette smoking* was when a participant smoked at least one cigarette/day at the time of this study, and smoking was categorized as “yes”, while not smoking was categorized as “no” [9]. *Consuming salt* was described in the questionnaire as consuming processed food mostly containing sodium, adding salt during cooking at home, and adding salt to food during mealtime; this was categorized into a “no” category, indicating not consuming salt; “yes”, indicating consuming salt; and “sometimes”, indicating occasionally consuming salt [54].

### 2.5. Data Analysis

STATA version 18 (StataCorp. 2023. Stata Statistical Software: Release 18. College Station, TX, USA: StataCorp LLC.) was used to analyse data. Missing data were assessed using complete case analysis. Uncontrolled hypertension was the dependent variable, and Chi square (χ^2^) and binary logistic regressions were used to identify its predictors. The covariates were socio-demographic variables, anthropometric measurements, selected behavioural and biological risk factors, and treatment variables. Variables that were found to have an association with the outcome variable at *p*-value < 0.25 in the bivariate regression analysis were entered to the multivariable logistic regression model for final modelling. The magnitude of association between independent and dependent variables was measured using odds ratios and a 95% confidence interval (CI) with significant levels (*p*-value < 0.05).

### 2.6. Ethics Statement

The Research and Ethics Committee, Sefako Makgatho Health Sciences University (SMUREC), approved this study (SMUREC/H/259/2020: PG). Further permission was obtained from the Department of Health in Gauteng Province (NHRD Ref no: 202103_035), and from the managers of the selected facilities. Patients gave informed consent for their participation, and this study adhered to ethical principles [55].

## 3. Results 

### 3.1. Clinical Characteristics of the Patients

With a response rate of 99%, a total of 400 chronic hypertensive out-patients participated in this study. The mean age of the patients was 50 ± 8 years, and the prevalence of uncontrolled hypertension (HTN) was estimated at 57%. Fifteen percent (15%) had been hospitalized due to hypertension-related ill-health, and diabetes (27%) was the commonest comorbidity. Approximately 52% of the patients self-reported having been diagnosed with hypertension for over 5 years at the time of this study. Almost all patients (98%) did not have devices to monitor their blood pressure levels at home, and a family history of hypertension was self-reported in 86% of the study population (Table 1).

### 3.2. Socio-Demographic Characteristics

Female patients were 232 (58%), and males were 168 (42%). Over half of the patients [*n* = 227 (55%)] were aged 50 years and older. The participants were black Africans living in peri-urban settings, and most of them came from poor socio-demographic backgrounds in terms of being single (43%), unemployed (61%), and living in larger households with five members and more (34%); almost half (46%) were living in households with income of up to USD 26,085/month (ZAR 5000/month) (Table 2).

### 3.3. Selected Modifiable Lifestyle Factors

#### 3.3.1. Comparison of Selected Modifiable Lifestyle Factors and Treatment by Blood Pressure Control Status

The prevalence of obesity (62% vs. 42%, *p* ≤ 0.0001), salt consumption (90% vs. 55%, *p* ≤ 0.0001), alcohol intake (42% vs. 19%, *p* ≤ 0.0001), smoking habits (23% vs. 4%, *p* ≤ 0.0001), alternative medicine use (51% vs. 40%, *p* = 0.043), and comorbidities (64% vs. 36%, *p* ≤ 0.0001) were higher in the uncontrolled group than the controlled group, whereas the prevalence of physical activity (38% vs. 15%, *p* ≤ 0.0001) was high in the controlled group vs. the uncontrolled. Bi- and tri-therapies were the most common, and BP control agents were classified based on their frequency of being mentioned in any of the therapies. A thiazide diuretic [TD; hydrochlorothiazide (70%)] was the most used control agent, followed by the calcium channel blocker [CCB; amlodipine (68%)] and angiotensin-converting enzyme inhibitor [ACE inhibitor; enalapril (42%)]; TD was significantly different between the controlled and uncontrolled HBP (*p* = 0.029). Overall, 85% of the patients moderately adhered to treatment, only 2% exhibited high adherence, and 13% demonstrated low adherence (Table 3).

#### 3.3.2. Predictors of Uncontrolled Hypertension (Logistic Regression Analysis)

Table 4 shows the association of uncontrolled hypertension with predictors. Categories in variables with a small number of cases were merged in a regression analysis. In univariable logistic regressions, uncontrolled hypertension was associated with BMI, WC, WHR, WHtR, education, physically active, current alcohol use, current cigarette smoking, salt consumption, hospital admission, adherence to treatment, and use of alternative medicines (*p* ≤ 0.25). After controlling for potential confounders (i.e., sociodemographic, behavioural, and biological variables), the final hierarchical logistic regression showed significant associations of uncontrolled hypertension with the number of prescribed antihypertensive therapies [adjusted odds ration = 2.39; 95% confidence interval: 1.48–3.87], adherence to treatment [0.46; 0.21–0.97], salt consumption [28.35; 7.87–102.04], being physically active [0.22; 0.13–0.37], current alcohol use [2.10; 1.22–3.61], and current cigarette smoking [4.79; 1.88–12.18].

## 4. Discussion

This study investigated uncontrolled hypertension, treatment, and predictors among hypertensive out-patients attending primary health care (PHC) facilities in Johannesburg, South Africa. Most participants were living in poor socioeconomic environments, which tend to predispose an individual to chronic diseases compared to the least deprived environments [56,57]. Participants living in poor environments use PHC facilities that are overcrowded, with long queues, and lack resources, such as medicine stock-outs, compared to private facilities [28,29,30]. Therefore, addressing social inequalities regarding healthcare use is critical to achieve better health outcomes for patients in public facilities [30]. Further, two-thirds of the patients with uncontrolled hypertension in this study had comorbidities, and diabetes was the commonest comorbidity, consistent with reports in some African countries [10,13]. The presence of many chronic diseases has been implicated in challenges of controlling hypertension [58], especially among patients with diabetic and kidney disease comorbidities [28,35]. Therefore, hypertensive patients do face a challenge of uncontrolled BP, due to the presence of comorbidities. 

A high prevalence rate of uncontrolled hypertension (57%) was estimated among hypertensive out-patients in this study. Although this prevalence is higher than the prevalence reported in the South African National Health and Nutrition Examination Survey (13.5%) [3], it is almost similar to several local studies conducted in different provinces such as in Kwa-Zulu Natal (51%) [59], and Mpumalanga (56.83%) [4], and it is lower compared to Eastern Cape (75.5%) [13]. Furthermore, a current analysis of blood pressure screening among 4727 South Africans (mean age 40.9 ± 18.1 years) showed that 31.9% had hypertension. Of those with hypertension, only 42.5% were aware, and 36.1% were receiving treatment for hypertension. A large proportion (48.5%) of individuals receiving antihypertensive medication had uncontrolled BP [60]. In comparison to other African and Asian countries, the prevalence in this study is similar in Ghana (57.7%) [61], and it is higher compared to studies conducted in Ethiopia (37%) [62] and Israel (35.9%) [63] but lower in India (63.6%) [64], Zimbabwe (67.2%) [10], the Democratic Republic of Congo (77.5%) [65], and Morocco (82.8% [66]. These variations could be due to the differences in the application of the operational definition of uncontrolled hypertension applied in this study and other studies [46,47,65]. Another reason could be the dissatisfaction with primary care services, especially in the public sector, which often leads many individuals to health care shop or to visit higher level hospitals for primary care, which consequently leads to considerable inefficiency and loss of control over efficacy and quality of services [28]. It is not surprising that the use of alternative medicine (46%), mainly consulting traditional healers, using herbal/traditional medicine, and/or prayer camps, was reported in this study and associated with uncontrolled hypertension (χ^2^ test and univariate analysis), similar to other studies [67,68]. 

Uncontrolled hypertension was more likely among patients on tri therapy compared to those on less than two therapies in this current study. The association of increasing the number of pills and uncontrolled hypertension can be explained as a reversed causality. This happens when BP does not respond to treatment; consequently, more classes are added to the treatment to lower BP, depending on the level of BP and response to medication [69]. Most hypertensive patients in this study were on either on two (i.e., bi) or three (i.e., tri) therapies, while very few were on a one (i.e., mono) and quartet treatment (four drugs). The most prescribed hypertensive medications used by the patients in this study were hydrochlorothiazide (a thiazide diuretic; TD), followed by enalapril (angiotensin-converting enzyme inhibitor; ACE inhibitor) and amlodipine (a calcium channel blocker; CCB). Researchers have acknowledged success in hypertension control through a combination of drug therapies [70]. Furthermore, the odds of uncontrolled hypertension were lower among patients who adhered (i.e., average) to antihypertensive medication compared to those who did not adhere (i.e., low), as previously reported [65,71]. Adherence to antihypertensive medications leads to controlling high BP via vasodilatation, increasing urination to remove excess salt and fluid from the body, which leads to decreasing BP [72,73]. 

Other predictors of uncontrolled hypertension in this study were salt consumption, being physically active, current alcohol use, and smoking cigarettes, as previously reported [10,13,47,58,74]. Over two-thirds of the patients in this study consumed salt (described as consuming processed food mostly containing sodium, adding salt during cooking at home, and using salt from tables set for mealtimes), which possibly contributed to higher odds of uncontrolled hypertension. This has been attributed to high-salt diets activating the renin–angiotensin system, causing sodium retention, which increases renal reabsorption and leads to elevated blood pressure [75]. It is worth noting that Regulation 214 (R214) of the Foodstuffs, Cosmetics and Disinfectants Act No. 54 of 1972 in 2013 in South Africa (i.e., salt legislation) was enforced on 30 June 2016, and further salt reductions were implemented from 30 June 2019 [76]. With that being said, preliminary findings suggest that South Africa’s salt regulation has been effective in lowering salt intake in young adults by ~1.2 g salt/day in a longitudinal study [77]. 

Again, patients who were physically active were less likely to have uncontrolled hypertension. Studies in South Africa, Ethiopia, and Zimbabwe [4,7,10,58] have reported similar results, which might be explained through the possible mechanism that physical activity controls high BP and prevents weight gain, as well as increases endothelial function and decreases psychosocial stress [78]. Furthermore, in line with our results, consuming alcohol has been associated with higher odds of uncontrolled hypertension in other LMICs [47]. Although the mechanism through which alcohol raises blood pressure remains unclear, stimulation of the renin–angiotensin–aldosterone system, increased cortisol levels, and increased vascular reactivity may contribute to hypertension [79]. Also, cigarette smoking was more likely to be associated with uncontrolled hypertension in this study, consistent with other reports substantially linking smoking and uncontrolled hypertension [10,47]. Further possibilities include increased peripheral vascular resistance due to high blood pressure caused by smoking, which leads to narrowed and damaged arteries, in addition to the stimulation of the sympathetic nervous system due to the nicotine in cigarettes [47]. This suggests that lifestyle modification is paramount to reduce and control for hypertension.

### Limitations

The interpretation of these current results is limited by the following points. This study used a cross-sectional design, which can only estimate well-founded inferences and rules out causality. Use of non-probability sampling could have introduced bias, which was mitigated by obtaining a larger sample size of participants. Recall bias, social desirability, and unmeasured confounders might have influenced factors associated with uncontrolled hypertension. Use of dichotomous and trichotomous responses for salt consumption, current alcohol use, cigarette smoking, and physical activity does not provide sufficient information on practices. Therefore, comprehensive and standardized tools that include quantitative measures, such as frequency, quantity, and types, should be considered in future studies, as well as a detailed investigation on comorbidities, including HIV status, and a comprehensive biochemical assessment related to uncontrolled blood pressure. Caution must be taken when generalizing the results of this study, especially in resource-constrained and context-specific settings. Nonetheless, our study has been able to investigate the prevalence, treatment, and predictors of uncontrolled hypertension among hypertensive out-patients attending primary health care facilities in Johannesburg, South Africa. The correspondence of the prevalence, adherence to treatment, and predictors in this study with other studies provides support to these current results. 

## 5. Conclusions

This study reports a high prevalence of uncontrolled hypertension accompanied by comorbidities among out-patients living in environments of poor socioeconomic status and attending selected public PHC facilities in Johannesburg, South Africa. This is irrespective of the common BP control agents (TD, CCB, and ACE inhibitors) used by patients to treat hypertension, with over half being on tri-therapy, over two-thirds having low to moderate treatment adherence, and almost none with high adherence. Predictors of uncontrolled hypertension are the number of therapies, adherence to treatment, salt intake, physical activity, current alcohol use, and current cigarette smoking. Worth noting is that higher odds of uncontrolled hypertension were more noticeable among patients consuming salt than other predictors. Amidst the legislation of reducing salt intake by the South African National Department of Health [80], it seems as though we are not meeting the recommendation, and the WHO has reported that salt intake in the country is double the recommended quantity per day [81]. The high prevalence of uncontrolled hypertension in this study suggests a need to optimize prescriptions, adherence to BP lowering, and lifestyle modification. Additionally, the management of comorbidities such as diabetes could offer considerable benefits in controlling blood pressure. 

## Figures and Tables

**Table 1:** Clinical characteristics of the patients.

Variables	Categories	Frequency (n)	Proportion (%)
Blood pressure status	Controlled Uncontrolled	173 227	43 57
Duration of hypertension	<5 years 5 to 10 years Above 10 years	192 103 105	48 26 26
Have BP cuff	No Yes	392 8	98 2
Family history of hypertension	No Yes	55 345	14 86
Presence of comorbidities	No Yes	192 208	48 52
Have diabetes	No Yes	292 108	73 27
Other cardiovascular diseases	No Yes	350 50	87 13
Hospitalized in the past 3 months. (Hypertension related)	No Yes	342 58	85 15

**Table 2:** Socio-demographic characteristics of the patients.

Variables	All *n* = 400 n (%)	Controlled HTN *n* = 227 n (%)	Uncontrolled HTN *n* = 173 n (%)	*p*
Sex				
Males	168 (42)	67 (39)	101 (44)	0.247
Females	232 (58)	106 (61)	126 (56)	
Age (years)				
<50	191 (48)	81 (47)	110 (48)	0.745
≥50	209 (52)	92 (53)	117 (52)	
Marital status				
Single	172 (43)	77 (45)	95 (42)	0.830
Cohabiting	28 (7)	11 (6)	17 (8)	
Married	143 (36)	63 (36)	80 (35)	
Widowed/divorced	57 (14)	22 (13)	35 (15)	
Education level				
No/primary	82 (21)	28 (16)	54 (24)	0.174
Secondary/Grade 12	269 (67)	123 (71)	146 (64)	
Post grade 12	49 (12)	22 (13)	27 (12)	
Employment status				
No	243 (61)	107 (62)	136 (60)	0.810
Yes	129 (32)	53 (31)	76 (33)	
Pensioner	28 (7)	13 (8)	15 (7)	
Household member				
<5	257 (64)	116 (67)	141 (62)	0.307
≥5	143 (36)	57 (33)	86 (38)	
Household income/month				
<USD 26,085 (ZAR 5000)	283 (71)	120 (69)	163 (72)	0.525
USD 26,085 (ZAR 5000)–USD 52,200 (ZAR 10,000)	72 (18)	30 (17)	42 (19)	
>USD 52,200 (ZAR R10,000)	45 (11)	3 (13)	22 (9)	
Electricity				
No	9 (2)	2 (1)	7 (3)	0.198
Yes	391 (98)	171 (99)	220 (97)	
Refrigerator use				
No	21 (5)	9 (5)	12 (5)	0.970
Yes	379 (95)	164 (95)	215 (95)	
Water access				
No	4 (1)	4 (2)	0 (0)	0.021
Yes	396 (99)	169 (98)	227 (100)	
Toilet type				
Pit	26 (6)	8 (5)	18 (8)	0.402
Flush	370 (93)	163 (94)	207 (91)	
Both	4 (1)	2 (1)	2 (1)	

**Table 3:** Comparison of modifiable lifestyle factors and treatment among patients by blood pressure status.

Variables	All	Controlled HTN	Uncontrolled HTN	*p*
BMI				
Underweight	4 (1)	1 (1)	3 (1)	≤0.0001
Normal	82 (21)	41 (24)	41 (18)	
Overweight	101 (25)	59 (34)	42 (19)	
Obesity	213 (53)	72 (42)	141 (62)	
WC				
Normal	23 (6)	13 (8)	10 (4)	0.186
Abdominal obesity	377 (94)	160 (92)	217 (96)	
WHR				
Normal	116 (29)	58 (34)	58 (26)	0.082
Abdominal obesity	284 (71)	115 (66)	169 (74)	
WHtR				
Normal	359 (90)	160 (92)	199 (87)	0.115
Abdominal obesity	41 (10)	13 (8)	28 (12)	
Salt consumption				
No	47 (11)	44 (25)	3 (1)	≤0.0001
Yes	300 (75)	95 (55)	205 (90)	
Sometimes	53 (13)	34 (20)	19 (8)	
Physically active				
No	301 (75)	107 (62)	194 (85)	≤0.0001
Yes	99 (25)	66 (38)	33 (15)	
Current alcohol use				
No	245 (61)	123 (71)	122 (54)	≤0.0001
Yes	127 (32)	32 (19)	95 (42)	
Sometimes	28 (7)	18 (10)	10 (4)	
Current cigarette smoking				
No	338 (85)	165 (95)	173 (76)	≤0.0001
Yes	59 (15)	6 (4)	53 (23)	
Sometimes	3 (1)	2 (1)	1 (1)	
Classes of BP control agents				
TD	281 (70)	128 (74)	153 (64)	0.153
Non-TD	119 (30)	45 (26)	74 (32)	
CCB	270 (68)	118 (68)	152 (67)	0.792
Non-CCB	130 (32)	55 (32)	75 (33)	
ACE inhibitor	168 (42)	62 (36)	106 (45)	0.029
Non-ACE inhibitor	232 (58)	111 (64)	121 (53)	
Number of prescribed antihypertensive therapy				
Mono therapy	3 (1)	2 (1)	1 (1)	0.0001
Bi-therapy	185 (46)	105 (61)	80 (35)	
Tri-therapy	197 (49)	60 (35)	137 (60)	
Quartet	15 (4)	6 (3)	9 (4)	
Adherence to treatment				
Low (<50%)	55 (13)	13 (8)	42 (19)	≤0.0001
Moderate (50–79%)	339 (85)	160 (92)	179 (79)	
High (≥80%)	6 (2)	0	6 (3)	
Alternative medicine use				
No	215 (54)	103 (60)	112 (49)	0.043
Yes	185 (46)	70 (40)	115 (51)	
Comorbidities				
No	192 (48)	111 (64)	81 (36)	≤0.0001
Yes	202 (52)	62 (36)	146 (64)	

TD stands for Thiazide diuretics, CCB stands for calcium channel blocker, ACE stands for angiotensin-converting enzyme, and a comorbidity entails the presence of one or more additional medical conditions or co-existing with hypertension.

**Table 4:** Predictors of uncontrolled hypertension.

Uncontrolled Hypertension	OR (95%CI)	*p*	AOR (95%CI)	*p*
Number of Therapies				
Bi	1		1	
Tri	2.92 (1.94–4.40)	≤0.0001	2.39 (1.48–3.87)	≤0.0001
Adherence to treatment				
Non-adherence	1		1	
Adherence	0.36 (0.19–0.69)	0.002	0.46 (0.21–0.97)	0.042
Physically active				
No	1		1	
Yes	0.28 (0.17–0.45)	≤0.0001	0.22 (0.13–0.37)	≤0.0001
Salt consumption				
No	1		1	
Yes	25.47 (7.76– 83.67)	≤0.0001	28.35 (7.87–102.04)	≤0.0001
Current alcohol use				
No	1		1	
Yes	2.12 (1.39–3.22)	≤0.0001	2.10 (1.22–3.61)	0.007
Current cigarette smoking				
No	1		1	
Yes	6.44 (2.97–13.94)	≤0.0001	4.79 (1.88–12.18)	0.001

## Data Availability

The dataset for the study group generated and analysed during this current study is available from the corresponding author upon reasonable request due to ethical restrictions.
